# Editorial: Mental health in recreational and elite sports

**DOI:** 10.3389/fpsyg.2025.1748059

**Published:** 2025-12-05

**Authors:** Renátó Tóth, Bettina Franciska Pikó, Tamás Berki, Martin James Turner, László Tóth

**Affiliations:** 1 School of Doctoral Studies, Hungarian University of Sports Science, Budapest, Hungary; 2 Department of Behavioral Sciences, University of Szeged, Szeged, Hungary; 3 Department of Physical Education Theory and Methodology, Hungarian University of Sports Science, Budapest, Hungary; 4 Faculty of Health and Education, Manchester Metropolitan University, Manchester, United Kingdom; 5 Institute of Teacher Training, Hungarian University of Sports Science, Budapest, Hungary; 6 Department of Psychology and Sport Psychology, Hungarian University of Sports Science, Budapest, Hungary

**Keywords:** mental health, recreational athletes, elite sport, physical activity, sport psychology

## Introduction

Mental health in sport has emerged as a central concern in contemporary research, reflecting the growing recognition that psychological wellbeing is a fundamental component of both athletic development and performance. Athletes across all levels and ages face a complex interplay of psychological, social, and physiological demands that influence their mental health trajectories. The aim of this Research Topic was to consolidate current empirical and theoretical contributions that deepen our understanding of these dynamics, while also identifying effective strategies for prevention, intervention, and support across diverse sporting populations. The Research Topic brings together studies that collectively highlight not only the prevalence and multifaceted nature of mental health challenges in sport, but also the expanding evidence-base for targeted psychological approaches designed to enhance performance, and overall wellbeing.

The articles accepted in this Research Topic can be organized into four overarching thematic categories: investigations of the relationship between physical activity and mental health, analyses of the distinctive psychological demands faced by elite athletes, examinations of mental health challenges among student athletes, and evaluations of psychological interventions aimed at promoting athlete mental health.

## Physical activity and mental health

Chang examined 903 Chinese international students in South Korea and found that physical activity was negatively associated with depression and positively related to self-efficacy, which partially mediated this relationship, while social support further moderated the effects of physical activity on depression. Similarly, Wang et al. reported high heterogeneity in depression-related outcomes, influenced by factors such as age, frequency, duration, and exercise period, yet their subgroup analyses consistently showed that physical exercise is associated with improvements in depressive symptoms, although it does not reduce suicide risk. Beyond identifying these associations, several studies also propose practical directions for intervention: Zhao R. et al. found that modern dance may be more effective in promoting recovery in individuals with depressive mood than Chinese classical, ballet, or jazz dance; and Garcia-Gullien et al. highlighted the importance of encouraging physical activity and balanced technology use to enhance psychosocial development and interpersonal skills in educational contexts. In addition, a cross-sectional study of 701 participants showed that exercise status did not significantly affect smartphone addiction (SA) or usage, although exercisers exhibited a weaker link between SA and screen time, suggesting more functional mobile phone use (Pirwani et al.). Other findings indicate that mental fatigue awareness reduces recovery, moderated by gender (Kara et al.), while recreational flow and wellbeing increased the likelihood of re-participation in sports Bayram et al.. Sahebi et al. further demonstrated that various sports can improve speech and social abilities in autistic children while also benefiting gut microbiota composition. Taken together, these studies underscore the substantial role of physical activity in supporting mental health, reinforcing the need for effective professional strategies to motivate individuals to engage in exercise. In this context, Turner et al. found that Rational Emotive Behavior Therapy (REBT) may enhance self-determined motivation, readiness, and confidence to participate in physical activity.

## Mental health of elite athletes

The scoping review by Jean-Bindley et al. provides a valuable synthesis of previous findings on prevention and intervention strategies for identifying and managing psychological distress. Resperger et al. and Nuetzel likewise highlight the importance of recognizing and addressing the diverse stressors athletes encounter throughout their careers. These stressors can profoundly affect the daily lives of professional athletes; for example, Gil-Caselles et al. found that psychological distress not only increases injury risk but may also prolong recovery time in triathletes. Konstantinides et al. concluded that marital satisfaction, poor health, and concerns about chronic traumatic encephalopathy contribute to caregiver burden. Complementing this, Deng et al. demonstrated that a growth mindset enhances competitive motivation by reducing stress responses and increasing basic psychological needs, with stronger effects observed among elite athletes. Further emphasizing individualized support needs (Busch et al.), using regression tree analysis, identified that current mood and stress as the strongest predictors of mental health among German Paralympic athletes facing physical health challenges. Nogueira et al. underscored that the way athletes interpret and channel emotions—not merely their intensity—plays a crucial role in competitive performance and psychological adaptation. Stress, anxiety, and mood states may also be closely associated with dropout; Kovács et al. found that dropout rates were higher in individual sports than in team sports. Adding a sport-specific perspective, Ihász et al. highlighted in a case study the extreme physical and emotional demands of SuperEnduro racing, suggesting that strong emotional attachment—rather than addiction—may underpin persistence and motivation in high-risk, high-intensity sports. Karoglu et al. found that handgrip strength relates differently to exercise addiction depending on sport type and sex among elite athletes. A crucial professional aspect was emphasized by Colangelo et al., who stressed the need for fairness in professional sport and proposed ways in which sport psychiatrists and sports medicine physicians can advocate for education among players, healthcare professionals, and governing bodies. Overall, the studies accepted in this Topic examined elite athletes through diverse mental health perspectives, and Scheer et al. further demonstrated the importance of identifying and screening for anxiety and sleep problems, as well as fostering awareness, education, preventative strategies, and support services.

## Student athletes' mental health

In addition to studies focusing on elite athletes, the Topic also included research involving university athletes. In a clinical trial among college students, Liu et al. found that the experiment group receiving peer support intervention demonstrated greater improvements than the control group receiving low-intensity mental health education, including enhanced psychological resilience, reduced perceived stress, increased positive moods (such as activity and calmness), and reduced negative moods (such as anxiety and depression). Complementing these findings, Ji and Li highlighted the potential of Tai Chi Chuan (TCC) to restore cognitive performance and reduce mental fatigue following prolonged cognitive effort amon university students. Beyond this populations, Clark et al. showed that high athletic identity in adolescent volleyball players was associated with greater anxiety and depression.

## Psychological interventions for athlete mental health

The studies presented above provide important empirical insights into the effects and predictors of different levels of sport participation on mental health. However, it is also essential to highlight those numerous contributions within this Topic examined the effectiveness of various (sport)psychological interventions. For example, Zhao J. et al. found that attentional bias correction training can effectively reduce pre-match anxiety and attentional bias toward negative information in high-level athletes. In a meta-analysis, Li et al. showed that psychological interventions—particularly traditional psychological skills training (PST)—significantly reduced athletes' anxiety. One of the most common PST tools was investigated by Zhang et al., who concluded that imagery offers psychological benefits for athletes, although it should be applied with caution, considering individual differences and potential limitations. Wood and Turner found that integrating traditional PST techniques (e.g., goal setting, imagery) with elements of Acceptance and Commitment Therapy (ACT) may be particularly effective in defusing unhelpful cognitions, reinforcing valued living and committed action, and enhancing present-moment focus. Moreover, by integrating polyvagal theory with performance models, Blanco and Tyler positioned vagal-centered approaches as promising, evidence-based strategies for optimizing athlete health and performance. Beyond applying appropriate interventions, it is equally important that mental health professionals operate in roles that align with best practice; in this regard, the guidelines proposed by James and Turner for professional soccer academies offer valuable direction that may be applicable across other sports as well.

## Conclusions

Collectively, the contributions to this Research Topic offer a comprehensive and multilayered perspective on mental health in sport, spanning general populations, elite performers, student athletes, and applied intervention research. Across these diverse domains, the findings converge on the recognition that mental health is not only integral to athletic performance but is also shaped by a complex interaction of psychological, social, and physiological factors that evolve across the lifespan and competitive levels. The evidence presented underscores both the protective potential of physical activity and the substantial mental health challenges that can emerge in high-performance contexts, highlighting the importance of early identification, tailored support, and the integration of mental health literacy within sporting environments. Studies involving student athletes similarly emphasize the need for preventive strategies account for developmental vulnerabilities and the dual pressures of academic and athletic demands. Equally important, the growing body of research on psychological interventions demonstrates the efficacy of diverse approaches—including attentional bias training, psychological skills training, ACT, REBT, and vagal-centered frameworks—in reducing distress and enhancing adaptive functioning in athletes when implemented within clear professional boundaries.

Together, these findings reinforce the critical need for interdisciplinary collaboration, evidence-informed practice, and organizational structures that prioritize mental health as a core component of athlete development and wellbeing. As the field continues to expand, future research should build on these foundations by addressing gaps in cross-cultural understanding, long-term intervention outcomes, and the integration of mental health support across all levels of sport.

